# Postpancreatectomy acute pancreatitis after distal pancreatectomy: tri-institutional international cohort

**DOI:** 10.1093/bjsopen/zrag069

**Published:** 2026-07-03

**Authors:** Elisa Romandini, Giampaolo Perri, Aya Maekawa, Elisa Bannone, Mushegh A Sahakyan, Dyre Berg Kleive, Poya Ghorbani, Giovanni Marchegiani, Roberto Montorsi, Alice Cattelani, Stefan Gilg, Åsmund Avdem Fretland, Marcus Holmberg, Ernesto Sparrelid, Knut Jørgen Labori, Bjørn Edwin, Roberto Salvia

**Affiliations:** Department of General and Pancreatic Surgery, Verona University Hospital, Verona, Italy; Department of Hepato-Pancreato-Biliary Surgery, Oslo University Hospital, Oslo, Norway; Department of Gastrointestinal Surgery, Hamar Hospital, Hamar, Norway; Department of Surgery, Oncology and Gastroenterology (DiSCOG), University of Padua, Hepato-pancreato-biliary and Liver Transplant Surgery, Padua, Italy; Division of Surgery, Department of Clinical Science, Intervention and Technology, Karolinska University Hospital, Stockholm, Sweden; Department of Surgery, Oncology and Gastroenterology (DiSCOG), University of Padua, Hepato-pancreato-biliary and Liver Transplant Surgery, Padua, Italy; Department of General and Pancreatic Surgery, Verona University Hospital, Verona, Italy; Department of Surgery, Vestre Viken Hospital Trust, Ringerike Hospital, Hønefoss, Norway; The Intervention Centre, Oslo University Hospital, Rikshospitalet, Oslo, Norway; Department of Surgery N1, Yerevan State Medical University, Yerevan, Armenia; Department of Hepato-Pancreato-Biliary Surgery, Oslo University Hospital, Oslo, Norway; Division of Surgery, Department of Clinical Science, Intervention and Technology, Karolinska University Hospital, Stockholm, Sweden; Department of Surgery, Oncology and Gastroenterology (DiSCOG), University of Padua, Hepato-pancreato-biliary and Liver Transplant Surgery, Padua, Italy; Department of General and Pancreatic Surgery, Verona University Hospital, Verona, Italy; Department of General and Pancreatic Surgery, Verona University Hospital, Verona, Italy; Division of Surgery, Department of Clinical Science, Intervention and Technology, Karolinska University Hospital, Stockholm, Sweden; Department of Hepato-Pancreato-Biliary Surgery, Oslo University Hospital, Oslo, Norway; The Intervention Centre, Oslo University Hospital, Rikshospitalet, Oslo, Norway; Division of Surgery, Department of Clinical Science, Intervention and Technology, Karolinska University Hospital, Stockholm, Sweden; Emergency, Upper GI, Bariatric and Abdominal Wall Surgery, St.Görans Hospital, Stockholm, Sweden; Division of Surgery, Department of Clinical Science, Intervention and Technology, Karolinska University Hospital, Stockholm, Sweden; Department of Hepato-Pancreato-Biliary Surgery, Oslo University Hospital, Oslo, Norway; Institute of Clinical Medicine, University of Oslo, Oslo, Norway; Department of Hepato-Pancreato-Biliary Surgery, Oslo University Hospital, Oslo, Norway; The Intervention Centre, Oslo University Hospital, Rikshospitalet, Oslo, Norway; Institute of Clinical Medicine, University of Oslo, Oslo, Norway; Department of General and Pancreatic Surgery, Verona University Hospital, Verona, Italy

**Keywords:** pancreatic surgery, left resection, complications, pancreatic fistula, hyperamylasaemia, PPAP

## Abstract

**Background:**

The International Study Group for Pancreatic Surgery (ISGPS) recently introduced a standardized definition and grading system for postpancreatectomy acute pancreatitis (PPAP), which was validated for pancreatoduodenectomy but not for distal pancreatectomy (DP). This study investigated the incidence and predictors of PPAP following DP and its associated postoperative complications.

**Methods:**

Patients who underwent DP for all indications between 2015 and 2022 at three European centres were retrospectively analysed. PPAP was defined according to the ISGPS criteria (postoperative serum hyperamylasaemia (POH), radiological findings) and patient clinical deterioration. Standard univariable and multivariable and receiver operating characteristic (ROC) curve analyses were conducted.

**Results:**

Among 1192 patients, PPAP occurred in 45 (3.8%): 35 grade B (2.9%) and 10 grade C (0.8%). POH without PPAP occurred in 165 patients (13.8%). Clinical outcomes worsened progressively across patients without POH/PPAP, with POH only, and with PPAP (Clavien–Dindo ≥ IIIa: 17.8%, 21.2%, and 53.3%, respectively; overall *P* < 0.001). PPAP was associated with higher rates of postoperative complications, including pancreatic fistula (68.9%), delayed gastric emptying (17.8%), and unplanned intensive care unit admission (17.8%). Compared with POH only, PPAP was associated with higher C-reactive protein (CRP) levels on postoperative day (POD) 2 and POD3. Among patients with POH, higher CRP levels on POD3 remained independently associated with PPAP in multivariable analysis (adjusted odds ratio 1.01; *P* < 0.001), together with multiorgan resection and smoking status. ROC curve analyses supported the discriminatory performance of CRP, particularly on POD3.

**Conclusion:**

PPAP is a rare but potentially severe complication after DP. Among patients with POH, elevated early postoperative CRP, particularly on POD3, may allow early risk stratification, identifying those at high risk of PPAP who may benefit from targeted mitigation strategies.

## Teaser

This large retrospective tri-institutional international analysis defines postpancreatectomy acute pancreatitis (PPAP) as a rare but severe complication that markedly worsens the postoperative course after distal pancreatectomy. Postoperative hyperamylasaemia and C-reactive protein were found to be reliable early biomarkers of PPAP that identify patients who may benefit from targeted mitigation strategies.

## Introduction

Postpancreatectomy acute pancreatitis (PPAP) has recently emerged as a distinct complication following major pancreatic resections, with the potential to adversely impact postoperative outcomes^1^. The clinical impact and sequelae of PPAP have been predominantly investigated after pancreatoduodenectomy^1–4^, highlighting its contribution to the development of several additional postoperative complications, particularly postoperative pancreatic fistula (POPF)^5^. Conversely, fewer studies with inconsistent metrics have explored PPAP after distal pancreatectomy (DP)^6–8^.

The definition and grading system of the International Study Group for Pancreatic Surgery (ISGPS) is based on the clinical impact of PPAP^1^. According to this definition, PPAP is an acute inflammatory condition of the pancreatic remnant that manifests early after partial pancreatic resection. Its diagnosis relies on the fulfilment of three criteria: postoperative hyperamylasaemia (POH), indicated by a sustained elevation of serum amylase activity exceeding the institutional upper limit of normal and persisting on at least postoperative days (POD) 1 and 2; association with a clinically relevant downturn in the patient's condition; and radiological findings consistent with PPAP.

Early recognition of PPAP may have critical clinical implications because it could enable the appropriate and timely management of postoperative adverse events, potentially averting a higher postoperative burden and clinical downturn^2–4,9^. However, differences between pancreatoduodenectomy and DP regarding PPAP remain unexplored. Distinct anatomical and surgical features, as well as variations in postoperative burden between these procedures, exist^10^. As for POPF and other pancreas-specific postoperative complications, the mechanisms triggering morbidity after DP are likely unique and deserve specific investigations to define possible mitigation strategies.

The aim of the present international multicentre study was to investigate the incidence of PPAP following DP by applying the ISGPS definition and grading system. In addition, the study explored postoperative complications associated with PPAP, and identified risk factors contributing to its occurrence.

## Methods

### Study design

This international multicentre retrospective study was conducted at three referral centres for pancreatic surgery. The study was approved by the respective institutional ethics committees and/or local data protection officers. The study adheres to the principles outlined in the Declaration of Helsinki and follows the STROBE statement guidelines^11^. The STROBE checklist is presented in *Table S1*.

### Inclusion and exclusion criteria

Consecutively patients who underwent DP for any indication between January 2015 and June 2022 were included in the study. A prospectively maintained and anonymized database was used for data collection. Patients with incomplete data on postoperative serum pancreatic amylase activity on POD1 and/or POD2 were excluded. The surgeries were performed at the Department of General and Pancreatic Surgery, University of Verona Hospital Trust (Verona, Italy), the Department of Hepato-Pancreato-Biliary Surgery, Rikshospitalet, Oslo University Hospital (Oslo, Norway), and the Department of Surgery, Karolinska University Hospital (Stockholm, Sweden).

### Procedures and data collection

Information on demographic, intraoperative, postoperative, and pathological features was collected. Preoperative data included age, body mass index, smoking status (ever/never), co-morbidities, neoadjuvant treatment, and presumptive pathological diagnosis. The presumptive diagnosis, based on preoperative imaging and/or biopsy when available, was classified as either pancreatic ductal adenocarcinoma or all other diagnoses.

Intraoperative data encompassed the type of approach (minimally invasive (including laparoscopic and robot-assisted) or open), spleen preservation according to the Kimura *et al.*^12^ or Warshaw^13^ techniques, vascular resection, estimated blood loss, and operative time. Vascular resection was defined as any resection with reconstruction of the portal venous system and/or coeliac axis resection as part of the Appleby or modified Appleby procedures. Multiorgan resection was defined as simultaneous resection of at least one additional organ other than the pancreas and spleen. Surgeries converted from a minimally invasive approach were classified as open.

DPs were classified based on the level of the transection line: isthmus for transection at the pancreatic neck; parenchyma sparing for transection to the left of the portal vein axis at the level of the pancreatic body tail; and extended for transection at the level of the gastroduodenal artery. Different methods, such as scalpel, mechanical stapler, or ultrasonic devices, were used for pancreatic stump management. When the pancreas was transected with a scalpel, subsequent selective suturing of the main pancreatic duct was performed. In procedures in which a stapler was used, triple-row stapling technology was uniformly applied across all centres, and a stepwise parenchymal-flattening technique with prolonged peri-firing compression was used^14^. When an ultrasonic dissector was used for pancreatic transection, the dissector was operated at the lowest vibration level throughout the dissection process.

Drains were used based on local practice and surgeon preference. When placed, a drain was positioned next to the pancreatic stump. Drain management and removal were based on the clinical course, drainage output, and amylase levels in the drainage fluid. Pancreatic stump mesh reinforcement and prophylactic perioperative steroids were not used. Somatostatin analogues, including octreotide, were not routinely given for prophylaxis but could be administered selectively by the treating team.

### Endpoints

The primary endpoint of the study was to assess the incidence of PPAP following DP. Secondary endpoints included characterizing the postoperative course of patients with PPAP *versus* those with POH only or without POH/PPAP, as well as evaluating potential predictors of PPAP. The clinical burden associated with POH and PPAP was explored by stratifying the study population into three groups: patients without POH/PPAP; patients with POH only; and patients with PPAP.

### Outcomes metrics

PPAP was defined according to the ISGPS definition as POH, characterized by an elevation in pancreas-specific serum amylase activity greater than the institutional upper limit of normal persisting within at least the first 48 hours after surgery (including POD1 and POD2), combined with radiological changes consistent with PPAP on postoperative computed tomography (CT), and a clinically relevant deterioration in the patient’s condition. Based on clinical course, PPAP was classified as either grade B (for mild to moderate complications) or grade C (for severe life-threatening complications). This classification was determined retrospectively after the completion of postoperative follow-up.

The institutional upper limit of normal pancreas-specific serum amylase activity was 52 U/l at the University of Verona Hospital Trust and 65 U/l at both Oslo and Karolinska university hospitals.

Contrast-enhanced CT imaging was performed as clinically indicated during the postoperative course, particularly in the event of clinical deterioration or suspected abdominal complications. Radiological findings of PPAP were assessed up to 30 days after surgery and were defined as reduced enhancement of the pancreatic stump, parenchyma swelling, and/or peripancreatic adipose tissue stranding. All CT scans were independently reviewed in a blinded manner by two experienced surgeons (G.P. & E.B.) or radiologists, with diagnostic uncertainties resolved by consensus and, if necessary, by a third expert.

No standardized treatment protocol was used for PPAP. Other pancreas-specific complications, including POPF^5^, postpancreatectomy haemorrhage (PPH)^15^, and delayed gastric emptying (DGE)^16^, were defined according to the ISGPS criteria. Organ space surgical site infection and respiratory, cardiovascular, thromboembolic, and urological complications were recorded as predefined clinically relevant events requiring specific treatment or intervention, and are detailed in the *supplementary methods*. Serum C-reactive protein (CRP) levels were measured on POD1, POD2, and POD3. Drain fluid amylase activity was assessed on POD1, POD3, and POD5 when drains were in place. Postoperative complications were classified using the Clavien–Dindo system^17^ over a 90-day period, with major complications defined as Clavien–Dindo grade IIIa or higher. Data on reoperation, length of hospital stay, and readmissions were retrieved from medical records. Intensive care unit (ICU) admission was defined as an unplanned requirement for intensive care. Readmission was defined as any unplanned hospital readmission within 30 days after surgery. Mortality was recorded as any postoperative death occurring within 90 days after surgery.

### Statistical analysis

Continuous variables are expressed as the median with interquartile range (i.q.r.) and were compared with the Mann–Whitney *U* test or the Kruskal Wallis test, as appropriate. Categorical variables are presented as absolute numbers with percentages and were compared with the χ^2^ test or Fisher's exact test, as appropriate. Two-sided *P* < 0.05 was considered significant for each test. A Holm–Sidak correction was applied for multiple comparisons.

Multivariable logistic regression models were fitted with PPAP as the dependent variable. In the overall cohort, clinically relevant preoperative and intraoperative variables with *P* < 0.10 in univariable analyses were considered for inclusion in the multivariable model. A separate univariable and multivariable model was also used in patients with POH to evaluate factors associated with progression to PPAP. Candidate preoperative and intraoperative variables, together with CRP on POD3, were assessed. To reduce collinearity, CRP concentrations on POD2 and POD3 were not included simultaneously in the same model.

The discriminative ability of CRP in predicting PPAP was assessed using receiver operating characteristic (ROC) curve analysis. The optimal threshold was determined through Youden index calculation.

Missing data were reported for each variable (*Table S2*). Analyses were performed using complete case analysis for the variables included in each model; no imputation was used, and denominators therefore vary across variables and models.

All statistical analyses were performed using SPSS^®^ version 22.0 (IBM, Armonk, NY, USA) and EZR (Saitama Medical Center, Jichi Medical University, Shimotsuke, Japan), a graphical user interface for R (version 4.2.5; R Foundation for Statistical Computing, Vienna, Austria).

## Results

### Baseline characteristics

**Fig. 1:**
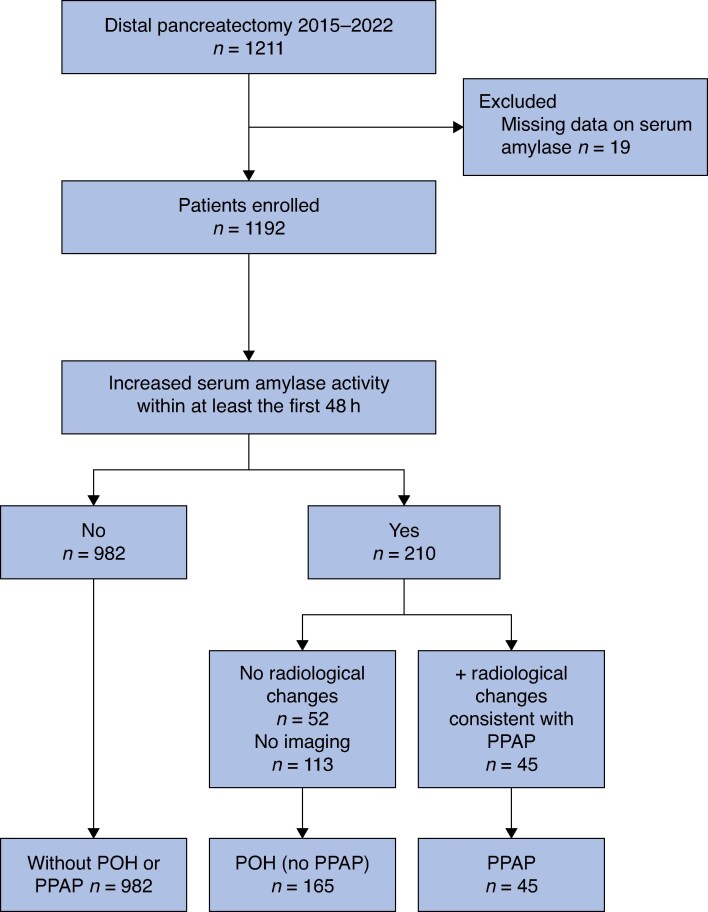
STROBE-compliant study flow chart POH, postoperative serum hyperamylasaemia; PPAP, postpancreatectomy acute pancreatitis; h, hours.

In all, 1192 patients were included in the analysis (*Fig. 1*). Baseline data, operative characteristics, and postoperative outcomes are summarized in *Table 1*.

**Table 1:** Patient characteristics (*n* = 1192)

**Sex**	
Male	562 (47.1%)
Female	630 (52.9%)
Age (years), median (i.q.r.)	65 (53–72)
BMI (kg/m^2^), median (i.q.r.)	25 (22–28)
Smoker	279 (23.4%)
Diabetes	232 (19.5%)
Cardiovascular disease	145 (12.2%)
COPD	110 (9.2%)
CKD	25 (2.1%)
ASA grade*	
I–II	866 (72.9%)
III–IV	322 (27.1%)
Neoadjuvant therapy	171 (14.3%)
**Pathological diagnosis**	
PDAC	495 (41.5%)
Others	697 (58.5%)
**Surgical approach**	
Open	634 (53.2%)
Laparoscopic	447 (37.5%)
Robotic	111 (9.3%)
Operative time (min), median (i.q.r.)	225 (167–300)
Estimated blood loss (ml), median (i.q.r.)	150 (50–350)
Spleen preserving	95 (8.0%)
Vascular resection	64 (5.4%)
Multiorgan resection	144 (12.1%)
**Transection line**	
Isthmus	799 (67%)
Parenchyma sparing	361 (30.3%)
Extended	32 (2.7%)
**Transection technique**	
Stapler	952 (80.2%)
Ultrasonic device	156 (13.1%)
Scalpel	79 (6.7%)
Drainless	25 (2.1%)
POH	210 (17.6%)
PPAP (clinically relevant)	45 (3.8%)
POPF (clinically relevant)	344 (28.9%)
**POPF grade**	
B	317 (26.6%)
C	27 (2.3%)
Organ space surgical site infection	457 (38.3%)
**Postpancreatectomy haemorrhage**	
Total	88 (7.4%)
Associated POPF (clinically relevant)	43 (3.6%)
Delayed gastric emptying	69 (5.8%)
Sepsis	67 (5.6%)
Respiratory morbidity	141 (11.8%)
Cardiac morbidity	54 (4.5%)
Urological morbidity	42 (3.5%)
Reoperation	61 (5.1%)
Unplanned ICU stay	56 (4.7%)
Mortality (90 days)	7 (0.6%)
LOS (days), median (i.q.r.)	8 (6–12)
Readmission (30 days)	171 (14.4%)
**Serum amylase (U/l), median (i.q.r.)**	
POD1	57 (36–87)
POD2	36 (23–54)
POD3	22 (14–33)
**CRP (mg/L), median (i.q.r.)**	
POD1	47 (28–73)
POD2	137 (90–196)
POD3	185 (127–259)
**Clavien–Dindo grade**	
0	354 (29.7%)
I	285 (23.9%)
II	321 (26.9%)
IIIa	124 (10.4%)
IIIb	56 (4.7%)
IVa	29 (2.4%)
IVb	16 (1.3%)
V	7 (0.6%)
Clavien–Dindo ≥ IIIa	232 (19.5%)

Values are *n* (%) unless otherwise stated. *Missing: *n* = 4. i.q.r., interquartile range; BMI, body mass index; COPD, chronic obstructive pulmonary disease; CKD, chronic kidney disease; ASA, American Society of Anesthesiologists; PDAC, pancreatic ductal adenocarcinoma; min, minutes; POH, postoperative serum hyperamylasaemia; PPAP, postpancreatectomy acute pancreatitis; POPF, postoperative pancreatic fistula; ICU, intensive care unit; LOS, length of hospital stay; POD, postoperative day; CRP, C-reactive protein.

### Clinical outcomes of PPAP after DP

Within the initial 48 h after DP, 210 patients (17.6%) exhibited elevated serum amylase activity. Among these patients, 45 (3.8% overall; 21.4% of patients with POH) developed characteristic radiological findings and clinically relevant complications, and were consequently classified as having PPAP. The remaining 165 patients had POH only (13.8%), of whom 52 had no radiological changes and 113 did not undergo imaging.

**Fig. 2:**
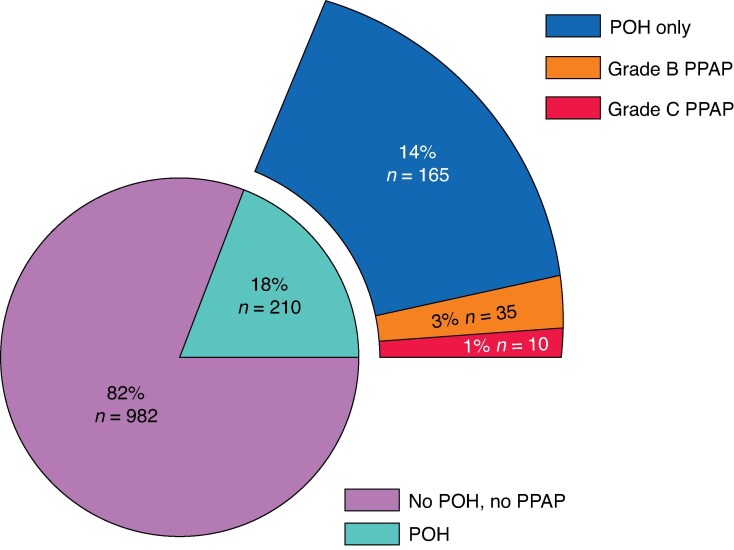
Population stratification based on the incidence of POH and PPAP POH, postoperative serum hyperamylasaemia; PPAP, postpancreatectomy acute pancreatitis.

Among patients diagnosed with PPAP, according to the ISGPS grading system^1^, 78% (35) were grade B and 22% (10) were grade C (*Fig. 2*). Postoperative outcomes stratified by PPAP grade are reported in *Table S3*. Clinically relevant POPF was frequent in both PPAP grades (65.7% in grade B *versus* 80.0% in grade C; *P* = 0.469). Grade C PPAP was associated with higher rates of unplanned ICU admission (50.0% *versus* 8.6%; *P* = 0.008) and 90-day mortality (20.0% *versus* 0%; *P* = 0.046) compared with grade B PPAP.

Postoperative outcomes stratified by the occurrence of POH and PPAP are presented in *Table 2*. The postoperative clinical course exhibited an increasing burden from patients without POH/PPAP to patients with POH only, culminating in the poorest outcomes for patients with PPAP. Specifically, severe postoperative morbidity escalated from 17.8% in patients without POH/PPAP to 21.2% in those with POH only and 53.3% in patients with PPAP (overall *P* < 0.001).

**Table 2:** Postoperative outcomes of the study cohort stratified by POH and PPAP occurrence

	No POH/PPAP (*n* = 982)	POH only (*n* = 165)	PPAP (*n* = 45)	*P*		
				Overall	No POH/PPAP *versus* POH	POH *versus* PPAP
**POPF (clinically relevant)**	254 (25.9%)	59 (35.8%)	31 (68.9%)	< 0.001	0.008	< 0.001
Grade B	241 (24.5%)	52 (31.3%)	24 (53.3%)	< 0.001	0.073	0.225
Grade C	13 (1.3%)	7 (4.4%)	7 (15.6%)			
**PPH**	57 (5.8%)	23 (13.9%)	8 (17.8%)	< 0.001	< 0.001	0.487
Grade B	32 (3.3%)	11 (6.7%)	5 (11.1%)	0.727	0.002	1.000
Grade C	8 (0.8%)	4 (2.4%)	2 (4.4%)			
**DGE**	55 (5.6%)	6 (3.8%)	8 (17.8%)	0.005	0.353	0.001
Grade B	13 (1.3%)	1 (0.6%)	4 (8.9%)	1.000	1.000	< 0.001
Grade C	7 (0.7%)	0	2 (4.4%)			
Organ space surgical site infection	341 (34.7%)	78 (47.3%)	38 (84.4%)	< 0.001	0.002	< 0.001
Sepsis	43 (4.4%)	16 (9.7%)	8 (17.8%)	< 0.001	0.004	0.183
Respiratory morbidity	88 (9%)	35 (21.2%)	18 (40%)	< 0.001	< 0.001	0.019
Cardiac morbidity	41 (4.2%)	10 (6.1%)	3 (6.7%)	0.371	0.305	1.000
Urological morbidity	28 (2.9%)	11 (6.7%)	3 (6.7%)	0.019	0.019	0.644
Reoperation	41 (4.3%)	14 (8.5%)	6 (13.3%)	0.004	0.028	0.389
Unplanned ICU stay	37 (3.8%)	11 (6.7%)	8 (17.8%)	< 0.001	0.092	0.036
Mortality (90 days)	3 (0.3%)	2 (1.2%)	2 (4.4%)	0.007	0.153	0.202
LOS (days), median (i.q.r.)	8 (6–11)	8 (7–14)	16 (11–26)	< 0.001	< 0.001	< 0.001
POD of last drain removal, median (i.q.r.)	5 (4–15)	7 (5–25)	23 (11–39)	< 0.001	0.004	0.001
Readmission (30 days)	129 (13.1%)	32 (19.4%)	10 (22.7%)	0.028	0.039	0.673
Clavien–Dindo ≥ IIIa	173 (17.6%)	35 (21.2%)	24 (53.3%)	< 0.001	0.275	< 0.001

Values are *n* (%) unless otherwise stated. POH, postoperative serum hyperamylasaemia; PPAP, postpancreatectomy acute pancreatitis; POPF, postoperative pancreatic fistula; PPH, postpancreatectomy haemorrhage; DGE, delayed gastric emptying; ICU, intensive care unit; LOS, length of hospital stay; i.q.r., interquartile range; POD, postoperative day.

Compared with patients without POH/PPAP and with POH only, those with PPAP exhibited significantly higher rates of pancreas-specific and systemic complications, in particular clinically relevant POPF (68.9%), as well as higher rates of major morbidities, unplanned ICU admission, and mortality (*Table 2*). Most patients with PPAP who developed a PPH had a concomitant clinically relevant POPF (7, 87.5%). PPAP was also significantly associated with prolonged hospital stays (median 16 (i.q.r. 11–26) days) and a higher 30-day readmission rate (22.7%; *Table 2*). All patients who developed PPAP had drains in place.

### Clinical outcomes of POH after DP

Patients with POH only exhibited worse outcomes compared with individuals without POH/PPAP. A CT scan was performed in 31.5% (52) of patients with POH (*versus* 15.0% (147) in the no POH/PPAP group), confirming the absence of PPAP.

Compared with patients without POH/PPAP, those with POH had significantly higher rates of clinically relevant POPF (35.8% *versus* 25.9%), PPH (13.9% *versus* 5.8%), organ space surgical site infections (47.3% *versus* 34.7%), and sepsis (9.7% *versus* 4.4%). Among patients with POH who developed PPH, 43.5% (10) had a concomitant clinically relevant POPF. POH was also associated with higher 30-day readmission rates (19.4% *versus* 13.1%). There was no significant difference between patients with and without POH in severe morbidity rates (21.2% *versus* 17.6%, respectively; *P* = 0.275) or 90-day mortality (1.2% *versus* 0.3%, respectively; *P* = 0.153). Among patients without POH/PPAP who underwent postoperative CT, radiological features consistent with pancreatitis were identified in 11%, despite an otherwise uncomplicated clinical course.

### Predictors of PPAP

Univariable analyses in the whole patient cohort identified a lower prevalence of smoking and higher body mass index in patients with PPAP. Patients who developed PPAP had significantly higher CRP levels compared with the POH group, with median values of 193 (i.q.r. 139–270) *versus* 147 (i.q.r. 101–204) mg/l on POD2 and 292 (i.q.r. 228–349) *versus* 218 (i.q.r. 152–289) mg/l on POD3 (*Table 3*).

**Table 3:** Preoperative, intraoperative, and early postoperative variables stratified by POH and PPAP occurrence after distal pancreatectomy

	No POH/PPAP (*n* = 982)	POH only (*n* = 165)	PPAP (*n* = 45)	*P*		
				Overall	No POH/PPAP *versus* POH	POH *versus* PPAP
**Sex**						
Male	465 (47.4%)	80 (48.5%)	17 (37.8%)	0.429	0.801	0.239
Female	517 (52.6%)	85 (51.5%)	28 (62.2%)			
Age (years), median (i.q.r.)	65 (54–72)	59 (49–69)	64 (52–70)	< 0.001	< 0.001	0.203
BMI (kg/m^2^), median (i.q.r.)	25 (22–28)	23 (21–26)	25 (23–28)	< 0.001	< 0.001	0.010
Smoker	227 (23.1%)	46 (27.9%)	6 (13.3%)	0.109	0.199	0.051
Diabetes	206 (21%)	19 (11.5%)	7 (15.6%)	0.011	0.005	0.452
Cardiovascular disease	131 (13.3%)	12 (7.3%)	2 (4.4%)	0.022	0.029	0.739
COPD	98 (10%)	9 (5.5%)	3 (6.7%)	0.158	0.081	0.723
CKD	14 (1.4%)	9 (5.5%)	2 (4.4%)	0.003	0.001	1.000
Non-PDAC pathological diagnosis	428 (43.6%)	52 (31.5%)	15 (33.3%)	0.007	0.004	0.858
Neoadjuvant therapy	153 (15.6%)	17 (10.3%)	1 (2.2%)	0.007	0.097	0.130
**ASA grade**						
I–II	688 (70.3%)	141 (86%)	37 (82.2%)	< 0.001	< 0.001	0.636
III–IV	291 (29.7%)	23 (14%)	8 (17.8%)			
**Surgical approach**						
Open	540 (55.0%)	75 (45.5%)	19 (42.2%)	0.001	< 0.001	0.658
Laparoscopic	366 (37.3%)	61 (37.0%)	20 (44.5%)			
Robotic	76 (7.7%)	29 (17.5%)	6 (13.3%)			
Spleen preserving	71 (7.2%)	19 (11.5%)	5 (11.1)	0.112	0.062	1.000
Vascular resection	54 (5.5%)	6 (3.6%)	4 (8.9%)	0.346	0.448	0.227
**Transection line**						
Isthmus	678 (69.0%)	98 (59.4%)	23 (51.1%)	0.009	0.073	0.315
Parenchyma sparing	277 (28.2%)	64 (38.8%)	20 (44.4%)			
Extended	27 (2.75)	3 (1.8%)	2 (4.4%)			
**Transection technique**						
Stapler	797 (81.5%)	124 (75.6%)	31 (68.9%)	0.003	0.002	0.323
Ultrasonic device	112 (11.5%)	34 (20.7%)	10 (22.2%)			
Scalpel	69 (7.1%)	6 (3.7%)	4 (8.9%)			
EBL (ml), median (i.q.r.)	150 (50–350)	200 (100–400)	200 (100–325)	0.074	0.067	0.600
Operative time (min), median (i.q.r.)	223 (163–300)	228 (183–297)	238 (175–300)	0.501	0.312	0.856
**CRP (mg/L), median (i.q.r.)**						
POD1	47 (28–73)	47 (27–77)	51 (34–86)	0.441	0.607	0.397
POD2	133 (85–190)	147 (101–204)	193 (139–270)	< 0.001	0.006	0.010
POD3	176 (122–248)	218 (152–289)	292 (228–348)	< 0.001	< 0.001	< 0.001
**Drain fluid amylase (U/l), median (i.q.r.)**						
POD1	1573 (571–3360)	3521 (1481–7500)	3475 (1808–6735)	< 0.001	< 0.001	0.878
POD3	249 (82–1017)	1129 (185–3126)	2223 (901–3344)	< 0.001	0.001	0.166
POD5	149 (37–1009)	501 (94–2734)	496 (71–7405)	0.0015	0.004	0.713

Values are *n* (%) unless otherwise stated. POH, postoperative serum hyperamylasaemia; PPAP, postpancreatectomy acute pancreatitis; i.q.r., interquartile range; BMI, body mass index; COPD, chronic obstructive pulmonary disease; CKD, chronic kidney disease; PDAC, pancreatic ductal adenocarcinoma; ASA, American Society of Anesthesiologists; EBL, estimated blood loss; min, minutes; CRP, C-reactive protein; POD, postoperative day.

**Fig. 3:**
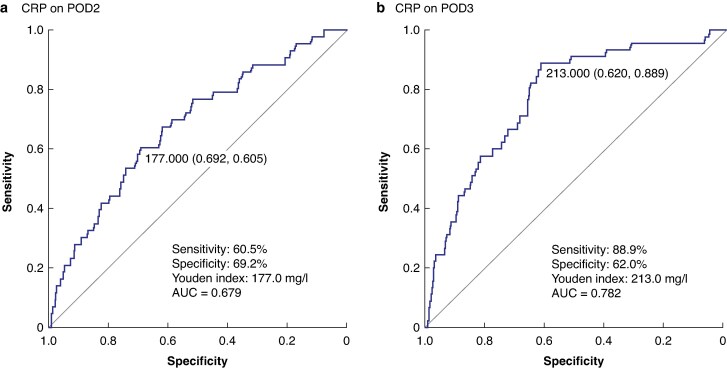
ROC curves of serum CRP for predicting postpancreatectomy acute pancreatitis in the overall cohort **a** ROC curve for CRP on POD2, with Youden index (95% c.i.); **b** ROC curve for CRP on POD3, with Youden index (95% c.i.). ROC, receiver operating characteristic; CRP, C-reactive protein; POD, postoperative day.

ROC curve analysis in the overall cohort indicated that CRP had moderate discriminative ability for PPAP, with an area under the curve (AUC) of 0.679 (95% confidence interval (c.i.) 0.595 to 0.762) on POD2 and 0.782 (95% c.i. 0.714 to 0.850) on POD3. The optimal CRP thresholds were 177.0 mg/l on POD2 (sensitivity 60.5%, specificity 69.2%) and 213.0 mg/l on POD3 (sensitivity 88.9%, specificity 62.0%; *Fig. 3*).

Among patients with POH, CRP on POD3 showed higher discriminative ability for PPAP than on POD2 (AUC 0.709 (95% c.i. 0.624 to 0.793) *versus* 0.628 (95% c.i. 0.532 to 0.725), respectively). The optimal CRP thresholds in this cohort were 193 mg/l on POD2 and 213 mg/l on POD3 (*Fig. S1*). In multivariable logistic regression limited to patients with POH, higher CRP on POD3 remained independently associated with PPAP after adjusting for selected clinical and operative covariates (adjusted odds ratio (OR) 1.01; 95% c.i. 1.00 to 1.01; *P* < 0.001; *Table 4*).

**Table 4:** Univariable and multivariable logistic regression analysis of factors associated with postpancreatectomy acute pancreatitis among patients with postoperative serum hyperamylasaemia

	Univariable		Multivariable	
	Odds ratio	*P*	Odds ratio	*P*
**Preoperative predictors**				
Age	1.01 (0.99–1.04)	0.227	1.00 (0.97–1.02)	0.795
Female sex	1.55 (0.79–3.05)	0.203		
Smoker	0.40 (0.16–1.00)	0.051	0.32 (0.12–0.89)	0.029
BMI ≥ 30 kg/m^2^	2.53 (0.98–6.54)	0.056	2.45 (0.88–6.81)	0.087
Diabetes	1.42 (0.55–3.61)	0.467		
Cardiovascular disease	0.59 (0.13–2.75)	0.505		
COPD	1.24 (0.32–4.78)	0.757		
CKD	0.81 (0.17–3.87)	0.784		
Non-PDAC pathological diagnosis	1.09 (0.54–2.19)	0.817		
Neoadjuvant therapy	0.20 (0.03–1.53)	0.120		
**Intraoperative predictors**				
Surgical approach				
Open	1.00 (Reference)			
Laparoscopic	1.29 (0.63–2.64)	0.478		
Robotic	0.82 (0.30–2.25)	0.695		
Spleen preservation	0.96 (0.34–2.73)	0.940		
Vascular resection	2.59 (0.70–9.59)	0.156		
Multiorgan resection	3.08 (1.26–7.51)	0.013	3.83 (1.42–10.40)	0.008
Transection line				
Isthmus	1.00 (Reference)			
Parenchyma sparing	1.33 (0.68–2.62)	0.407		
Extended	2.84 (0.45–18.00)	0.268		
Transection technique				
Stapler	1.00 (Reference)			
Ultrasonic device	1.00 (0–inf.)	1.000		
Scalpel	1.00 (0–inf.)	1.000		
Intraoperative blood loss (per 100 ml)	1.03 (0.96–1.11)	0.383		
Operative time (per 30 min)	1.00 (0.89–1.11)	0.936		
CRP (mg/L)				
POD1	1.00 (0.99–1.01)	0.714		
POD2	1.01 (1.00–1.01)	0.007		
POD3	1.01 (1.00–1.01)	< 0.001	1.01 (1.00–1.01)	< 0.001

Values in parentheses are 95% confidence intervals. BMI, body mass index; COPD, chronic obstructive pulmonary disease; CKD, chronic kidney disease; PDAC, pancreatic ductal adenocarcinoma; inf., infinity; min, minutes; CRP, C-reactive protein; POD, postoperative day.

In the overall cohort, multivariable logistic regression analysis identified multiorgan resection (OR 2.87; 95% c.i. 1.35 to 6.11; *P* = 0.006) and the use of an ultrasonic device for pancreatic transection (OR 2.65; 95% c.i. 1.24 to 5.66; *P* = 0.012) as intraoperative independent predictors of PPAP. Conversely, neoadjuvant therapy (OR 0.11; 95% c.i. 0.02 to 0.85; *P* = 0.034) was independently associated with lower PPAP risk (*Table 5*).

**Table 5:** Univariable and multivariable logistic regression analysis of preoperative and intraoperative predictors for postpancreatectomy acute pancreatitis in the overall study cohort

	Univariable		Multivariable	
	Odds ratio	*P*	Odds ratio	*P*
**Preoperative predictors**				
Age	0.99 (0.97–1.01)	0.701		
Female sex	1.43 (0.77–2.46)	0.251		
Smoker	0.50 (0.21–1.20)	0.126		
BMI ≥ 30 kg/m^2^	1.22 (0.56–2.66)	0.622		
Diabetes	0.77 (0.34–1.76)	0.545		
Cardiovascular disease	0.33 (0.08–1.39)	0.133		
COPD	0.57 (0.21–2.33)	0.575		
CKD	2.32 (0.53–10.20)	0.262		
Non-PDAC pathological diagnosis	0.71 (0.38–1.35)	0.310		
Neoadjuvant therapy	0.13 (0.02–0.95)	0.045	0.11 (0.02–0.85)	0.034
**Intraoperative predictors**				
Surgical approach				
Open	1.00 (Reference)			
Laparoscopic	1.52 (0.80–2.88)	0.202		
Robotic	1.85 (0.72–4.74)	0.200		
Spleen preservation	1.50 (0.57–3.91)	0.400		
Vascular resection	1.81 (0.62–5.23)	0.292		
Multiorgan resection	2.16 (1.05–4.46)	0.038	2.87 (1.35–6.11)	0.006
Transection line				
Isthmus	1.00 (Reference)			
Parenchyma sparing	1.98 (1.07–3.65)	0.029	1.81 (0.96–3.42)	0.067
Extended	2.25 (0.51–9.98)	0.286	2.00 (0.42–9.56)	0.384
Transection technique				
Stapler	1.00 (Reference)			
Ultrasonic device	2.03 (0.98–4.24)	0.058	2.65 (1.24–5.66)	0.012
Scalpel	1.58 (0.55–4.61)	0.398	1.55 (0.49–4.86)	0.454
Intraoperative blood loss (per 100 ml)	1.04 (0.99–1.09)	0.148		
Operative time (per 30 min)	1.00 (0.92–1.10)	0.918		

Values in parentheses are 95% confidence intervals. BMI, body mass index; COPD, chronic obstructive pulmonary disease; CKD, chronic kidney disease; PDAC, pancreatic ductal adenocarcinoma; min, minutes.

## Discussion

In this international multicentre retrospective study, the incidence of PPAP after DP overall was as low as 4%. However, patients with PPAP experienced a significantly higher rate of postoperative major morbidity (Clavien–Dindo ≥ IIIa), POPF, DGE, and unplanned ICU stay than those not developing PPAP. Postoperative outcomes worsened across patients from those without POH/PPAP, to those with POH only, and those who developed PPAP, supporting the clinical relevance of the progression from POH to PPAP. The present analysis complements a previous report^9^ on POH and clinically relevant POPF after DP by applying the ISGPS PPAP definition and grading system, and assessing PPAP-related outcomes.

Early postoperative CRP provided an informative signal associated with PPAP. Increased CRP levels in the early postoperative days were associated with the transition from POH towards a clinically relevant PPAP. Patients who developed PPAP had higher levels of CRP during POD1–3 than patients developing POH only. ROC analyses supported the discriminatory performance of CRP, with numerically better performance on POD3 than POD2. Importantly, in a multivariable model restricted to patients with POH, higher CRP on POD3 remained independently associated with progression to PPAP after adjustment for selected covariates. Together, the combination of biomarkers such as CRP and serum amylase levels enables the identification of a population with a very high risk of postoperative morbidity. These findings underscore the importance of early risk stratification, because such patients may represent ideal candidates for the application of mitigation strategies aimed at reducing the impact of PPAP and other pancreas-specific complications.

Grade B PPAP after DP was more common than grade C PPAP. Although less frequent, grade C PPAP was associated with a higher clinical burden, including increased unplanned ICU admission and 90-day mortality, compared with grade B (*Table S3*). Another relevant clinical scenario identified in the present analysis is represented by POH. This appears to be a common entity, affecting approximately one-fifth of patients undergoing DP. Although its occurrence is correlated with more severe complications *versus* patients without POH/PPAP, the presence of POH only, without PPAP, has not been associated with increased postoperative mortality. As in pancreatoduodenectomy, the present series confirms the progressive increase in postoperative complications from POH to PPAP. POH stands as the earliest identifiable serum marker of a complicated clinical course and could be a valuable tool for identifying individuals at increased risk of subsequent morbidity, as already stated in a recent tri-institutional study^9^. These patients may require a closer clinical surveillance and may not benefit from a fast-track approach in their postoperative course.

When observed in association with signs of initial clinical deterioration, POH combined with rising CRP levels requires a proactive approach to radiological imaging to prevent the underdiagnosis of an impending PPAP. Specific mitigation strategies following DP have not been standardized yet, so those already applied to pancreatoduodenectomy are normally used. These include dietary restriction, the use of somatostatin analogues, antibiotic therapy in the presence of infection, and prolonged drain maintenance when intraoperative drains were placed^18–24^. In a scenario in which the non-inferiority of omitting routine intra-abdominal drainage after DP on postoperative morbidity is established^22^, the need for early predictors of clinical outcomes becomes critical, and performing a CT scan may trigger the placement of a percutaneous drain in the event that fluid collection is detected. In this context, the combination of POH with elevated CRP may support early risk stratification to guide closer surveillance and timely imaging when clinically indicated. These considerations are in line with the available evidence, including a randomized clinical trial on pancreatoduodenectomy^19,20^. Importantly, because pancreas-specific complications tend to occur simultaneously, patients at risk of PPAP are also at higher risk of developing additional morbid events including POPF, PPH, and DGE. Therefore, preventing the escalation from POH to PPAP could act as a strategy to avoid or mitigate the overall burden of pancreas-specific complications.

This study has several limitations. First, PPAP was defined according to ISGPS criteria; however, these criteria do not specify the timing of postoperative CT, and both radiological and clinical components may be challenging to standardize retrospectively. Radiological criteria may require further refinement following DP. Second, postoperative CT was not systematically performed in patients with POH only, because 69% had no subsequent CT, which may have resulted in underdetection of PPAP and an underestimation of its true incidence. Third, the limited number of PPAP events increased the risk for type II error.

The strengths of the present study include the use of a large tri-institutional international cohort. This study identifies POH and CRP as effective early biomarkers of a complicated clinical course following DP. Notably, unlike pancreatoduodenectomy, DP involves far fewer confounding factors, given the absence of pancreatic, biliary, and intestinal anastomoses, which may serve as portals for infectious agents. From this perspective, data on POH and PPAP after DP may be considered purer and potentially more informative than corresponding data following pancreatoduodenectomy.

In conclusion, PPAP after DP is rare but associated with significantly worse postoperative outcomes than in patients without PPAP. POH represents the earliest recognizable biomarker of PPAP, and identifies patients with a potentially higher risk of clinical deterioration, especially if associated with increased CRP levels in the early postoperative days. This patient group is most likely to benefit from a proactive approach to postoperative imaging and the implementation of mitigation strategies.

## Supplementary Material

zrag069_Supplementary_Data

## Data Availability

The data are not publicly available due to privacy restrictions, but are available from the corresponding author upon reasonable request.
