# Supplementary material for: Postpancreatectomy acute pancreatitis after distal pancreatectomy: tri-institutional international cohort
Source: BJS Open. 2026 Jul 3;10(4):zrag069. doi: 10.1093/bjsopen/zrag069 (PMC13330926; doi:10.1093/bjsopen/zrag069)
Supplement: zrag069_Supplementary_Data [file zrag069_supplementary_data.docx]

**Postpancreatectomy Acute Pancreatitis after Distal Pancreatectomy: A Tri-institutional International Cohort**

Elisa Romandini MD^1,2,3^, Giampaolo Perri MD^4,5^, Aya Maekawa MD^4^, Elisa Bannone MD^1^, Mushegh A. Sahakyan MD^6,7,8^, Dyre Berg Kleive MD^2^, Poya Ghorbani MD^5^, Giovanni Marchegiani MD^4^, Roberto Montorsi MD^1^, Alice Cattelani MD^1^, Stefan Gilg MD^5^, Åsmund Avdem Fretland MD^2,7^, Marcus Holmberg MD^5,9^, Ernesto Sparrelid MD^5^, Knut Jørgen Labori MD^2,10^, Bjørn Edwin MD^2,7,10^, Roberto Salvia MD^1^

1 Verona University Hospital, Department of General and Pancreatic Surgery (Verona, Italy).

2 Oslo University Hospital, Department of Hepato-Pancreato-Biliary Surgery (Oslo, Norway).

3 Department of Gastrointestinal Surgery, Hamar Hospital (Norway)

4 University of Padua, Department of Surgery, Oncology and Gastroenterology (DiSCOG), Hepato-pancreato-biliary and Liver Transplant Surgery (Padua, Italy).

5 Karolinska University Hospital, Division of Surgery, Department of Clinical Science, Intervention and Technology (Stockholm, Sweden).

6 Department of Surgery, Vestre Viken Hospital Trust, Ringerike Hospital, Hønefoss, Norway.

7 The Intervention Centre, Oslo University Hospital, Rikshospitalet, Oslo, Norway.

8 Department of Surgery N1, Yerevan State Medical University, Yerevan, Armenia.

9 St. Görans Hospital, Emergency, Upper GI, Bariatric and Abdominal Wall Surgery (Stockholm, Sweden).

10 Institute of Clinical Medicine, University of Oslo, Norway

**Corresponding author.**

Giampaolo Perri, MD, PhD

Hepato Biliary Pancreatic (HPB) and Liver Transplant Surgery

Padova University Hospital, Via Giustiniani 2, 35128 Padova

**Supplementary Materials - Index**

| **Supplementary Methods** |  |
| --- | --- |
| Definitions of postoperative complications | *page 3* |
| **Supplementary Figures and Tables** |  |
| Supplementary Figure 1. | *page 4* |
| Supplementary Table 1. STROBE checklist. | *page 5-12* |
| Supplementary Table 2. Missing data by study variable. | *page 13* |
| Supplementary Table 3. Postoperative outcomes stratified by PPAP grade. | *page 14* |
| **References** | *page 15* |
| Reference for Supplementary Methods |  |

**Supplementary Methods**

**Definitions of postoperative complications**

Postoperative complications were recorded within 90 days following surgery, unless otherwise specified.

**Surgical site infection (SSI)**
Organ space SSI was defined as an infection involving any part of the anatomy other than the incision (organs or spaces) that was opened or manipulated during surgery, according to the National Surgical Quality Improvement Program definitions (1).

**Sepsis**
Sepsis was defined as a suspected or confirmed infection requiring escalation of care and/or intravenous antibiotic therapy.

**Respiratory complications**
Respiratory complications included any of the following events:

1. Pneumonia, diagnosed clinically or radiologically, and requiring antibiotic therapy.
2. Pleural effusion, requiring therapeutic drainage (thoracentesis or chest tube insertion).
3. Reintubation for acute respiratory failure requiring invasive ventilatory support.

**Thromboembolic complications**
Thromboembolic complications included clinically relevant venous thromboembolism requiring therapeutic anticoagulation, including pulmonary embolism.

**Cardiovascular complications**
Cardiovascular complications included any of the following events:

1. Atrial fibrillation with rapid ventricular response, requiring pharmacological treatment or electrical cardioversion.
2. Myocardial infarction diagnosed clinically, by electrocardiographic findings, or by biomarker evidence.
3. Myocardial dysfunction requiring inotropic support.
4. Cardiac arrest.

**Urologic complications**
Urologic complications included any of the following events:

1. Urinary tract infection diagnosed clinically and requiring antibiotic therapy.
2. Acute kidney injury, defined as postoperative deterioration of renal function documented in the medical record and requiring specific management.

**Supplementary Figures and Tables**

**Supplementary Figure 1.** Receiver operating characteristic curves of serum C-reactive protein for predicting postpancreatectomy acute pancreatitis among patients with postoperative hyperamylasaemia


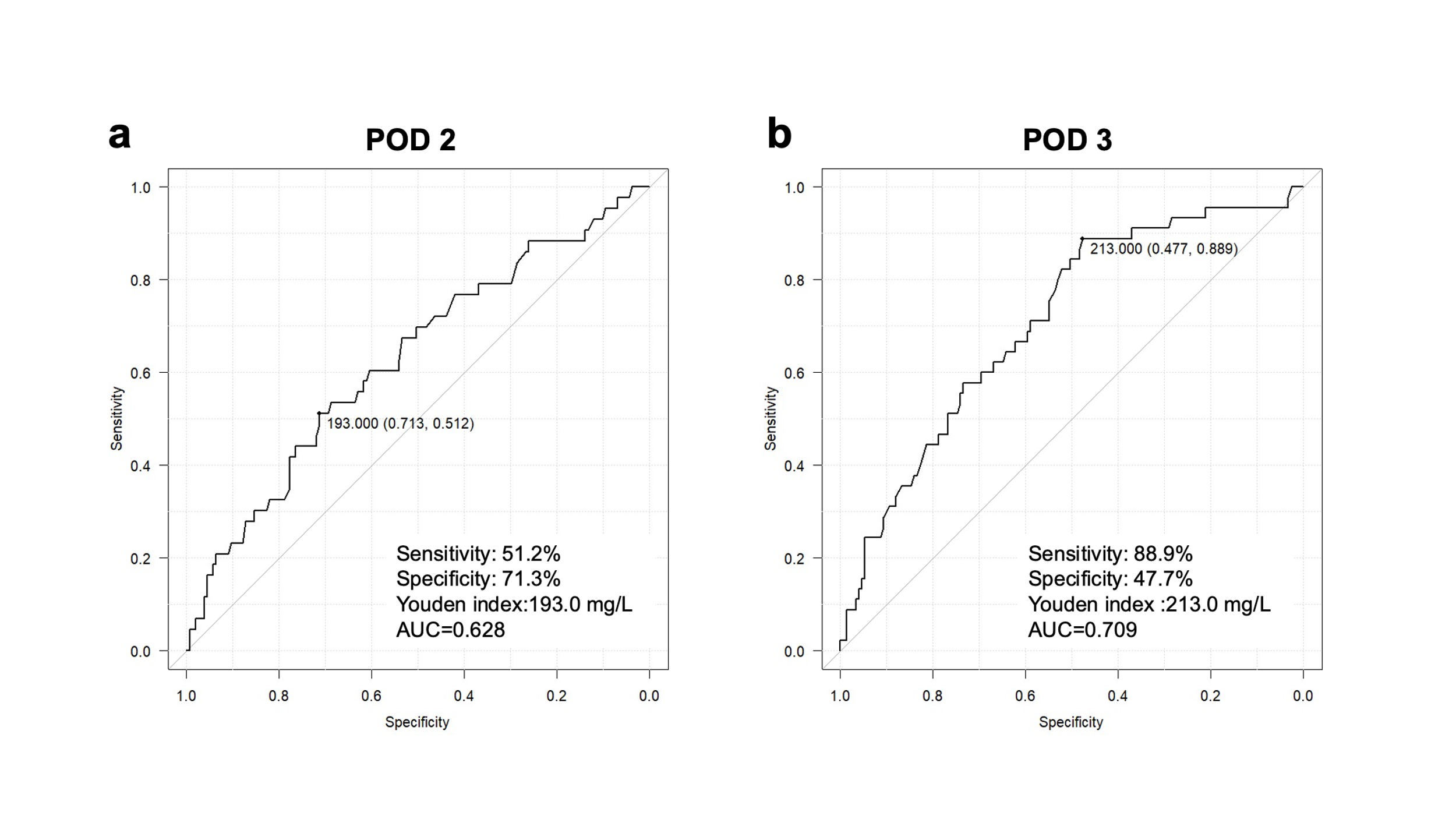


Supplemantary Table 1. STROBE checklist of items that should be included in reports of observational studies

|  | Item No. | Recommendation | Page No. | Relevant text from manuscript |
| --- | --- | --- | --- | --- |
| **Title and abstract** | 1 | (*a*) Indicate the study’s design with a commonly used term in the title or the abstract | 1, 3 | Title includes "Tri-institutional International Cohort"; Abstract methods describe a retrospective analysis at three centres. |
|  |  | (*b*) Provide in the abstract an informative and balanced summary of what was done and what was found | 3 | Abstract provides background, methods, results (incidence and outcomes), and conclusion. |
| Introduction | | | |  |
| Background/rationale | 2 | Explain the scientific background and rationale for the investigation being reported | 4 | Introduction describes PPAP after pancreatic resection, limited DP data, and rationale for applying ISGPS definition after DP. |
| Objectives | 3 | State specific objectives, including any prespecified hypotheses | 4 | Objective stated: to investigate incidence of PPAP after DP using ISGPS criteria and explore associated complications and predictors. |
| Methods | | | |  |
| Study design | 4 | Present key elements of study design early in the paper | 5 | Study design: "international multicentre retrospective study" at three referral centres; ethics approval; STROBE adherence. |
| Setting | 5 | Describe the setting, locations, and relevant dates, including periods of recruitment, exposure, follow-up, and data collection | 5 | Setting and dates: consecutive DP between Jan 2015 and Jun 2022 at Verona (Italy), Oslo (Norway), and Stockholm (Sweden). |
| Participants | 6 | (*a*) *Cohort study*—Give the eligibility criteria, and the sources and methods of selection of participants. Describe methods of follow-up  *Case-control study*—Give the eligibility criteria, and the sources and methods of case ascertainment and control selection. Give the rationale for the choice of cases and controls  *Cross-sectional study*—Give the eligibility criteria, and the sources and methods of selection of participants | 5, 7-8 | Eligibility: consecutive DP for any indication; excluded if POD1-2 serum amylase insufficient for POH determination; postoperative outcomes assessed up to 90 days from records. |
|  |  | (*b*) *Cohort study*—For matched studies, give matching criteria and number of exposed and unexposed  *Case-control study*—For matched studies, give matching criteria and the number of controls per case | NA | Not applicable (no matching). |
| Variables | 7 | Clearly define all outcomes, exposures, predictors, potential confounders, and effect modifiers. Give diagnostic criteria, if applicable | 6-8 | Variables defined: primary endpoint PPAP incidence; secondary endpoints include outcomes by POH/PPAP strata and predictors; diagnostic criteria for PPAP, POPF, PPH, DGE and others specified. |
| Data sources/ measurement | 8* | For each variable of interest, give sources of data and details of methods of assessment (measurement). Describe comparability of assessment methods if there is more than one group | 5-7 | Data sources and measurement: prospectively maintained anonymised databases; CT performed when clinically indicated; CT findings assessed up to 30 days and independently reviewed in blinded fashion by two experts with consensus process. |
| Bias | 9 | Describe any efforts to address potential sources of bias | 5, 7, 13 | Bias mitigation: consecutive inclusion; blinded independent CT review; limitations acknowledge potential underdetection because CT not systematic in POH only and retrospective standardisation challenges. |
| Study size | 10 | Explain how the study size was arrived at | 5, 9 | Study size: all eligible consecutive cases during study period included; final analytic cohort n=1192 (referenced in Results and Figure 1). |

Continued on next page

| Quantitative variables | 11 | Explain how quantitative variables were handled in the analyses. If applicable, describe which groupings were chosen and why | 8 | Quantitative variables handled as medians (IQR) and compared with non-parametric tests; continuous predictors entered as continuous terms in logistic regression; ROC-derived thresholds reported. |
| --- | --- | --- | --- | --- |
| Statistical methods | 12 | (*a*) Describe all statistical methods, including those used to control for confounding | 8 | Statistical methods: Mann-Whitney / Kruskal-Wallis; chi-square / Fisher exact; Holm-Sidak correction; multivariable logistic regression to assess predictors. |
|  |  | (*b*) Describe any methods used to examine subgroups and interactions | 6, 8 | Subgroup analyses: stratification into no POH/PPAP, POH only, PPAP; separate multivariable model restricted to patients with POH (PPAP vs POH only). |
|  |  | (*c*) Explain how missing data were addressed | 8 | Missing data: reported per variable (Supplementary Table 2); complete-case analysis for each model; no imputation; denominators vary. |
|  |  | (*d*) *Cohort study*—If applicable, explain how loss to follow-up was addressed  *Case-control study*—If applicable, explain how matching of cases and controls was addressed  *Cross-sectional study*—If applicable, describe analytical methods taking account of sampling strategy | NA | Not applicable (retrospective postoperative cohort; no loss to follow-up described). |
|  |  | (*e*) Describe any sensitivity analyses | NA | No sensitivity analyses reported. |
| Results | | | | |
| Participants | 13* | (a) Report numbers of individuals at each stage of study—eg numbers potentially eligible, examined for eligibility, confirmed eligible, included in the study, completing follow-up, and analysed | 5, 9 | Participants by stage: inclusion and exclusion criteria described in Methods; Results report n=1192 included (Figure 1). |
|  |  | (b) Give reasons for non-participation at each stage | 5, 9, 13 | Reasons for non-participation: excluded if POD1-2 amylase insufficient; Discussion notes CT not systematic in POH only which may affect PPAP ascertainment. |
|  |  | (c) Consider use of a flow diagram | 9, 18 | Flow diagram: Figure 1 (STROBE flowchart) referenced in Results; legend provided in Figures legend section. |
| Descriptive data | 14* | (a) Give characteristics of study participants (eg demographic, clinical, social) and information on exposures and potential confounders | 9 (Table I), 5-6 | Descriptive data: baseline characteristics, operative characteristics, and outcomes summarised in Table I; variables collected are specified in Methods. |
|  |  | (b) Indicate number of participants with missing data for each variable of interest | 8 (Suppl Table 2) | Missing data counts indicated in Supplementary Table 2; Methods state missing data are reported for each variable. |
|  |  | (c) *Cohort study*—Summarise follow-up time (eg, average and total amount) | 7-8 | Follow-up period defined for outcomes: complications classified over 90 days; readmission within 30 days; mortality within 90 days. |
| Outcome data | 15* | *Cohort study*—Report numbers of outcome events or summary measures over time | 9-11 (Table II-III) | Outcome events: incidence of POH and PPAP reported; complication rates and outcomes by POH/PPAP strata reported (Table II) and PPAP grade (Table S3). |
|  |  | *Case-control study—*Report numbers in each exposure category, or summary measures of exposure | NA | Not applicable (not a case-control study). |
|  |  | *Cross-sectional study—*Report numbers of outcome events or summary measures | NA | Not applicable (not a cross-sectional study). |
| Main results | 16 | (*a*) Give unadjusted estimates and, if applicable, confounder-adjusted estimates and their precision (eg, 95% confidence interval). Make clear which confounders were adjusted for and why they were included | 10-11 (Table IV-V) | Main results: univariable and multivariable logistic regression with ORs and 95% CI for predictors; POH subgroup model reports adjusted association of POD3 CRP with PPAP. |
|  |  | (*b*) Report category boundaries when continuous variables were categorized | 10, 18 | Category boundaries: continuous predictors analysed as continuous; ROC analysis reports optimal CRP thresholds (177 mg/L POD2, 213 mg/L POD3) derived by Youden index. |
|  |  | (*c*) If relevant, consider translating estimates of relative risk into absolute risk for a meaningful time period | NA | Not applicable (no translation of relative effects to absolute risk over time). |

Continued on next page

| Other analyses | 17 | Report other analyses done—eg analyses of subgroups and interactions, and sensitivity analyses | 9-11, 18 | Other analyses: ROC analyses for CRP on POD2 and POD3; additional ROC among POH patients (Supplementary Figure 1); outcomes stratified by PPAP grade and POH/PPAP groups. |
| --- | --- | --- | --- | --- |
| Discussion | | | | |
| Key results | 18 | Summarise key results with reference to study objectives | 11-12 | Key results summarised in Discussion with reference to objectives, including PPAP incidence after DP and association with worse morbidity and early CRP signal. |
| Limitations | 19 | Discuss limitations of the study, taking into account sources of potential bias or imprecision. Discuss both direction and magnitude of any potential bias | 13 | Limitations discussed: retrospective application of ISGPS criteria, non-standardised CT timing, CT not systematic in POH only leading to potential underdetection, limited PPAP events. |
| Interpretation | 20 | Give a cautious overall interpretation of results considering objectives, limitations, multiplicity of analyses, results from similar studies, and other relevant evidence | 13-14 | Interpretation and conclusion: PPAP after DP is rare but severe; POH and elevated early CRP may support early risk stratification and timely imaging; need for prospective studies. |
| Generalisability | 21 | Discuss the generalisability (external validity) of the study results | 13 | Generalisability: strengths include large tri-institutional international cohort; discussion notes DP-specific context and applicability to DP postoperative management. |
| Other information | | | | |
| Funding | 22 | Give the source of funding and the role of the funders for the present study and, if applicable, for the original study on which the present article is based | 1, 14 | Funding: "Source of funding: None" on title page; no acknowledgements; authors declare no conflicts of interest. |

*Give information separately for cases and controls in case-control studies and, if applicable, for exposed and unexposed groups in cohort and cross-sectional studies.

**Note:** An Explanation and Elaboration article discusses each checklist item and gives methodological background and published examples of transparent reporting. The STROBE checklist is best used in conjunction with this article (freely available on the Web sites of PLoS Medicine at http://www.plosmedicine.org/, Annals of Internal Medicine at http://www.annals.org/, and Epidemiology at http://www.epidem.com/). Information on the STROBE Initiative is available at www.strobe-statement.org.

| **Supplementary Table 2. Missing data by study variable.** | |
| --- | --- |
| **Variables** | **Missing N (%)** |
| ASA score | 4 (0.34%) |
| Operative time (min) | 5 (0.42%) |
| Estimated blood loss (mL) | 289 (24.2%) |
| Unplanned ICU admission, n (%) | 3 (8.6%) |
| Transection technique | 5 (0.42%) |
| Serum amylase POD 1 (U/l, median, IQR) | 41 (3.4%) |
| Serum amylase POD 2 (U/l, median, IQR) | 97 (8.1%) |
| Serum amylase POD 3 (U/l, median, IQR) | 206 (17.3%) |
| C–reactive protein POD 1 (mg/L, median, IQR) | 69 (5.8%) |
| C–reactive protein POD 2 (mg/L, median, IQR) | 45 (3.8%) |
| C–reactive protein POD 3 (mg/L, median, IQR) | 63 (5.3%) |
| Drain Fluid Amylase POD 1 (U/L) | 78 (6.54%) |
| Drain Fluid Amylase POD 3 (U/L) | 695 (58.3%) |
| Drain Fluid Amylase POD 5 (U/L) | 780 (65.4%) |
| POD of last drain removal | 46 (3.86%) |
| Length of hospital stay (days, median, IQR) | 3 (0.25%) |
| Readmission (30-day) | 2 (0.17%) |
| *Abbreviations: PPAP: postpancreatectomy acute pancreatitis; POPF: postoperative pancreatic fistula; PPH: post pancreatectomy haemorrhage; DGE: delayed gastric emptying; ICU: intensive care unit; IQR: interquartile range.* | |
| Percentages are calculated over the full cohort (N = 1192).  Drain fluid amylase values were available only when drains were in situ; missingness therefore reflects absence of drains or early drain removal. | |

| **Supplementary Table 3. Postoperative outcomes stratified by PPAP grade.** | | | | |
| --- | --- | --- | --- | --- |
|  | All PPAP  (n = 45) | Grade B  (n = 35) | Grade C  (n = 10) | P |
| POPF (Grade B/C), n (%) | 31 (68.9%) | 23 (65.7%) | 8 (80.0%) | 0.469 |
| PPH (Grade B/C), n (%) | 7 (15.6%) | 4 (11.4%) | 3 (30.0%) | 0.172 |
| DGE (Grade B/C), n (%) | 6 (13.3%) | 3 (8.6%) | 3 (30.0%) | 0.113 |
| Unplanned ICU admission, n (%) | 8 (17.8%) | 3 (8.6%) | 5 (50.0%) | 0.008 |
| Reoperation, n (%) | 6 (13.3%) | 3 (8.6%) | 3 (30.0%) | 0.113 |
| Mortality (90-days), n (%) | 2 (4.4%) | 0 (0%) | 2 (20.0%) | 0.046 |
| Length of hospital stay (days, median, IQR) | 16 (11.8–25.3) | 16 (11.0–25.5) | 18 (18.0–25.0) | 0.390 |
| *Abbreviations: PPAP: postpancreatectomy acute pancreatitis; POPF: postoperative pancreatic fistula; PPH: post pancreatectomy haemorrhage; DGE: delayed gastric emptying; ICU: intensive care unit; IQR: interquartile range.* | | | | |

**References**

1. Lawson EH, Hall BL, Ko CY. Risk factors for superficial vs deep/organ-space surgical site infections: implications for quality improvement initiatives. JAMA Surg. 2013;148(9):849-58.
